# solGS: a web-based tool for genomic selection

**DOI:** 10.1186/s12859-014-0398-7

**Published:** 2014-12-14

**Authors:** Isaak Y Tecle, Jeremy D Edwards, Naama Menda, Chiedozie Egesi, Ismail Y Rabbi, Peter Kulakow, Robert Kawuki, Jean-Luc Jannink, Lukas A Mueller

**Affiliations:** Boyce Thompson Institute for Plant Research, Cornell University, Ithaca, NY USA; National Root Crops Research Institute (NRCRI), Umudike, Nigeria; International Institute of Tropical Agriculture (IITA), Ibadan, Oyo State Nigeria; National Crops Resources Research Institute, Kampala, Uganda; USDA-ARS, Robert W. Holley Center for Agriculture and Health, Cornell University, Ithaca, NY USA

**Keywords:** Genomic selection, RR-BLUP, Bioinformatics, Database, Web-based tool

## Abstract

**Background:**

Genomic selection (GS) promises to improve accuracy in estimating breeding values and genetic gain for quantitative traits compared to traditional breeding methods. Its reliance on high-throughput genome-wide markers and statistical complexity, however, is a serious challenge in data management, analysis, and sharing. A bioinformatics infrastructure for data storage and access, and user-friendly web-based tool for analysis and sharing output is needed to make GS more practical for breeders.

**Results:**

We have developed a web-based tool, called solGS, for predicting genomic estimated breeding values (GEBVs) of individuals, using a Ridge-Regression Best Linear Unbiased Predictor (RR-BLUP) model. It has an intuitive web-interface for selecting a training population for modeling and estimating genomic estimated breeding values of selection candidates. It estimates phenotypic correlation and heritability of traits and selection indices of individuals. Raw data is stored in a generic database schema, Chado Natural Diversity, co-developed by multiple database groups. Analysis output is graphically visualized and can be interactively explored online or downloaded in text format. An instance of its implementation can be accessed at the NEXTGEN Cassava breeding database, http://cassavabase.org/solgs.

**Conclusions:**

solGS enables breeders to store raw data and estimate GEBVs of individuals online, in an intuitive and interactive workflow. It can be adapted to any breeding program.

**Electronic supplementary material:**

The online version of this article (doi:10.1186/s12859-014-0398-7) contains supplementary material, which is available to authorized users.

## Background

Genomic selection (GS) is a new breeding paradigm that promises higher accuracy in estimating breeding values and a higher rate of gain from selection per unit time for complex quantitative traits compared to phenotypic selection or traditional marker assisted selection (MAS) [1-3]. GS was conceived with the advent of high-throughput technologies for whole genome sequencing, genotyping and identifying genetic variation in individuals [1,4]. Plant and animal breeders are finding GS appealing due to the progressive decline in genotyping cost and easier access to genome-wide markers such as single nucleotide polymorphism (SNP) markers, which can be genotyped using SNP array or genotyping-by-sequencing (GBS; [5]) technologies. However, the huge amount of data on which GS relies is challenging in its management, analysis and accessibility. The computational infrastructure and bioinformatics expertise GS requires are beyond the reach of a typical breeding program. A data management system and user-friendly web-based tool for GS analysis would add efficiency to the breeding decision-making process and make GS more accessible for breeders.

Genomic selection is a type of MAS. Individuals are genotyped with dense, genome-wide markers, such as SNPs, and phenotyped for traits of interest. This set of individuals, also called a training population, is used to create a genomic prediction model. A model estimates the sum of the additive genetic effects of the genome-wide alleles on the trait of individuals, referred to as, genomeic estimated breeding values (GEBVs). In selection cycles, individuals are genotyped, with the same set of markers as the training set, and the prediction model is used to predict their GEBVs for the trait of interest. Superior performers are selected based on their GEBVs and advanced to the next cycle of selection. Thus, skipping the phenotyping step of the same traits evaluated in the training set during selection cycles [1,6], which saves time.

An important step in the GS model fitting is the validation of its accuracy, which should be done before selecting candidates based on GEBVs. The most common approach is to estimate the correlation between GEBVs and observed phenotypes of individuals in the validation set [7]. A validation set can comprise of 10 - 30% of random individuals from the training set [7-9]. The GEBVs of the validation set are estimated using a model created based on the rest of the training set. 10 or more fold cross-validation tests are done; each time the validation set contains different individuals.

Genomic selection is being applied in animal and plant breeding programs. As early as 2001, animal breeders were experimenting, initially using simulation, with GS on dairy cattle traits [1,10,11]. Currently, it is also being tested or applied in plants such as maize [9], wheat [12], sugar beet [13] and cassava [14]. Hayes et al. [10] have extensively reviewed GS in animals. Nakaya and Isobe [3] also have documented a long list of animal and plant breeding programs where GS was used, including the traits, marker and population details, statistical methods used for the GS models and their accuracy.

GS is superior to MAS for complex traits, which is due to large scale of genome-wide markers capturing QTLs with medium and small effects [1]. GS experiments now typically include hundreds of individuals genotyped for thousands of markers. A maize GS experiment used 504 individuals genotyped for 158,281 SNP markers [9] and another wheat experiment used lines genotyped for 34,749 SNP markers [12]. The NEXTGEN Cassava project is genotyping hundreds of clones with up to 13,000 SNP markers (http://cassavabase.org).

The data intensive nature of this approach poses a computational challenge in terms of infrastructure for data storage. It has a high demand for expertise in data management, statistical analysis workflow, accessibility of results and data sharing. Furthermore, the complexity of GS statistical analysis is insufficiently understood by breeders [3]. Flexible GS databases and user-friendly web-based analytical tools would advance GS application in breeding programs [3,6].

solGS is a web-based tool that aims to address the bioinformatics and statistical challenge in GS. Its intuitive and user-friendly web-interface allows breeders to create prediction models and apply the model to predict GEBVs of selection candidates. It displays data graphically and interactively on a browser and also has options to download output into one’s computer. It uses an organism-agnostic database schema to store phenotype and genotype data, as well as experimental metadata [15]. The statistical modeling is based on the Ridge Regression Best Linear Unbiased Predictor (rrBLUP) R package [8]; GBLUP (genomic relationship matrix) method is used to estimate GEBVs.

solGS is, currently, used by the NEXTGEN Cassava project (http://nextgencassava.org) and implemented at the Cassavabase website (http://cassavabase.org/solgs). Here, we describe solGS using its implementation at the website using cassava data.

## Implementation

### Software

solGS is developed using open source software and runs on a Debian-based Linux server. For data storage, it uses a generic, organism-agnostic, relational Chado database schema, called Natural Diversity (ND) [15] on a PostgreSQL system (http://www.postgresql.org/, V., 9.1). The schema is ontology driven and is designed to store large-scale genotype, phenotype, and experimental data. For statistical analyses, it uses R [16] and specifically nlme (V. 3.1; [17] for the phenotype data preprocessing and rrBLUP (V., 3.8; [8]) for the statistical modeling. The application is developed on Catalyst Model-View-Controller (MVC) web framework [18] and is mostly in Perl. Mason templates are used for display layout, whereas JavaScript, including jQuery (http://jQuery.com), D3 (http://d3js.org) and Flot Chart (http://flotcharts.org) libraries are used for client-side user interactivity and graphical visualization of data. The web tool is compatible on all major browsers including FireFox, Safari, Chrome, and Internet Explorer.

### Data curation

Phenotype, genotype and experimental data are described with controlled vocabularies developed by curators in consultation with breeders; a reference for cassava trait ontology is at http://www.cropontology.org/ontology/CO_334/Cassava. In the current implementation, a curator also loads both phenotype and genotype data into the database, since the data may require preprocessing such as quality control, data clean up, ontology annotation in the case of phenotype data, and rigorous imputation in the case of genotype data. Accepted encodings for genotypes are [−1, 0, 1] and [0, 1, 2]. Considering the large size and complexity of the phenotype and genotype datasets and the need for experimental metadata, a fair amount of correspondence between curators and data providers is required. The loading scripts, as well as the rest of the code, are publicly available.

### Statistics

Prior to the prediction model fitting, phenotype data are preprocessed as follows: for individuals evaluated in randomized complete block design (RCBD), alpha lattice and augmented incomplete block designs, genotype effects are calculated using nlme R package’s lme function [17]. Genotypes are fit in the model as fixed effects whereas replications and/or blocks are fit as random effects. The model is fit by restricted maximum likelihood (REML). When trials have multiple phenotypic values per individual, for example when replications are completely randomized or no experimental design was stored in the database for the dataset, or multiple trial datasets were combined, an arithmetic mean for the individual is used. Individuals with missing phenotype values are omitted.

Currently, when multiple trials are selected to combine individuals and create a training population, first genotype effects or arithmetic phenotype mean, depending on the trial design, are calculated for the individuals within each trial. Then the genotype effects or arithmetic means are averaged across trials to create a single phenotype value for each individual.

By default, all missing marker values are imputed using K-Nearest Neighbors (KNN) method, from the Imputation R package (V., 1.3; http://cran.r-project.org/src/contrib/Archive/imputation/). However, we have not tested the effect of this method on the accuracy of a model. It is recommended that missing marker values are imputed prior to loading the marker data to the database, as is now practiced on Cassavabase.

The genomic prediction modeling is univariate and based on Ridge Regression Best Linear Unbiased Predictor (RR-BLUP) method [1], as implemented in the rrBLUP package [8]. The mixed.solve function, a linear mixed-model equation estimates marker effects and GEBVs. GEBVs are derived from the realized (additive) relationship matrix of individuals calculated from marker genotypes. The kinship.BLUP function, GBLUP, which uses mixed.solve, is called to predict GEBVs of selection candidates. Given preprocessing of phenotypes, a simple linear model for RR-BLUP applies:

$$ \mathrm{y}=\upmu +\mathrm{g}+\upvarepsilon $$

$$ \mathrm{g}\sim \mathrm{N}\left(\mathbf{0},\mathbf{K}{\sigma}_{\mathrm{g}}^2\right) $$

$$ \varepsilon \sim N\left(0,\kern0.5em I{\sigma}_{\varepsilon}^2\right) $$

Where is the vector of preprocessed phenotypes, *μ* is the population mean, *g* is the vector of genetic values, and *ε* is the vector of residuals. K is the additive (realized) relationship matrix calculated from marker genotypes. ${\sigma}_g^2$ and ${\sigma}_{\varepsilon}^2$ are the additive genetic and error variances, respectively. The vector of genetic values is the sum of the additive genetic random effects and is assumed to follow a normal distribution. From these parameters, narrow-sense heritability *h*^2^ [19] is calculated using

$$ {h}^2=\frac{\sigma_g^2}{\sigma_g^2+{\sigma}_{\varepsilon}^2} $$

To estimate model accuracy, a 10-fold cross-validation is performed. The training dataset is randomly divided into 10 equal sets or folds. In ten separate analyses, each fold is used as the validation set while the remaining nine folds are used to train the model. A correlation analysis between the GEBVs and the observed phenotype values of the validation sets is performed and the average correlation value of the 10 tests estimates the model accuracy.

### Usage

solGS is web-based and runs on a central server. After loading the relevant datasets into the database, breeders need only an Internet connection to access the tool, which in this case is hosted at http://cassavabase.org/solgs. An intuitive and user-friendly workflow guides breeders to perform the GS modeling, validation, and prediction of GEBVs. With essentially mouse-based input, breeders proceed through the workflow, visualize and download the results. Below, we demonstrate the analysis workflow with three use cases: trait approach, trial approach, and custom list approach (Additional file 1).

### Use case 1: Trait approach

#### Creating a prediction model

*This approach can be useful, for example, when breeders are about to initiate a breeding program to improve certain traits for a target environment and want to identify breeding material with superior breeding values for the traits to use in parental selection. Assume also that they have no prior knowledge of the trials or locations the traits were evaluated.*

**Figure 1:**
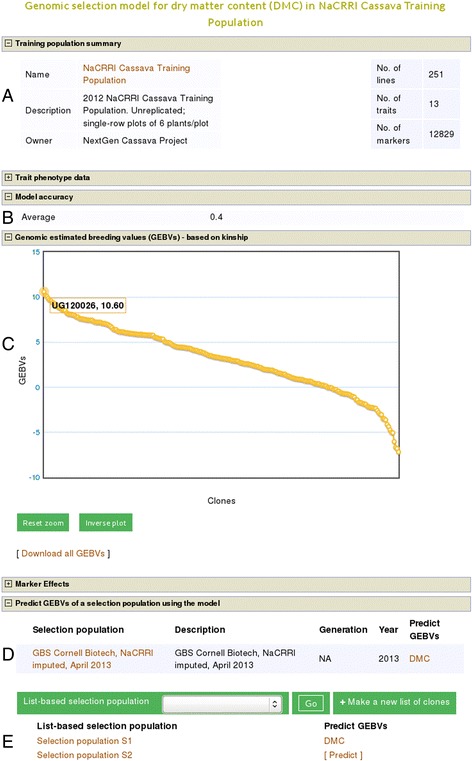
**Example of single prediction model output.** A single trait model output includes model summary **(A)**, a graphical representation of the phenotype data (collapsed; Figure 2), model accuracy **(B)**, the GEBVs of individuals in the training population **(C)**, and marker effects (collapsed). From the same model page, breeders can apply the model to predict GEBVs of selection populations **(D, E)**. GEBVs can be viewed in the browser using interactive graphs and be downloaded in text format.

**Figure 2:**
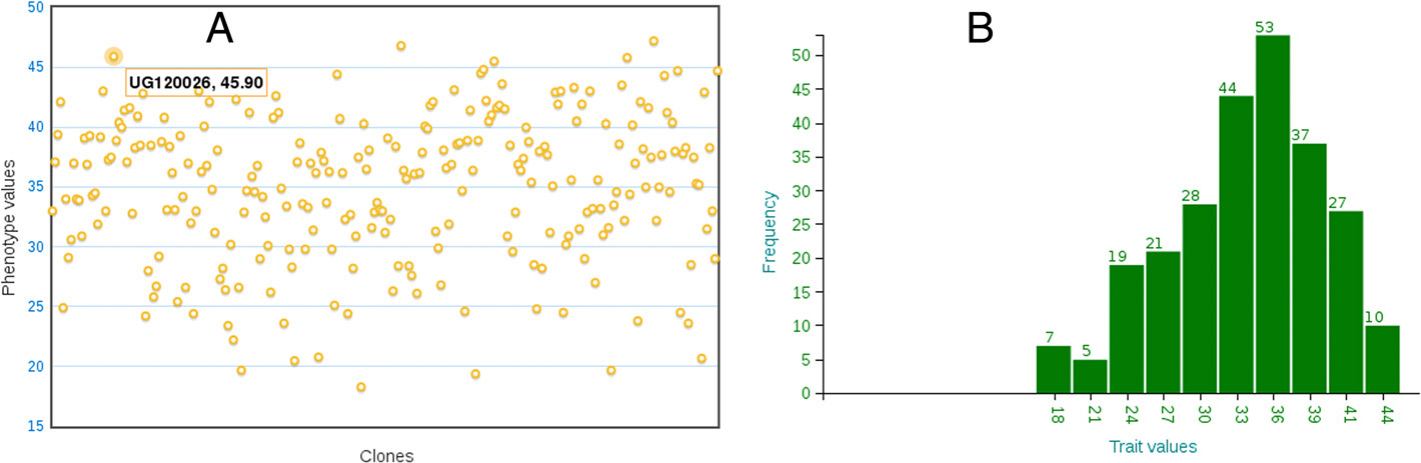
**Graphical representation of phenotype data used in a model.** Panel **A** shows an example interactive scatter plot of the phenotype data used in the model, where as panel **B** displays the frequency distribution of the same phenotype data.

**Figure 3:**
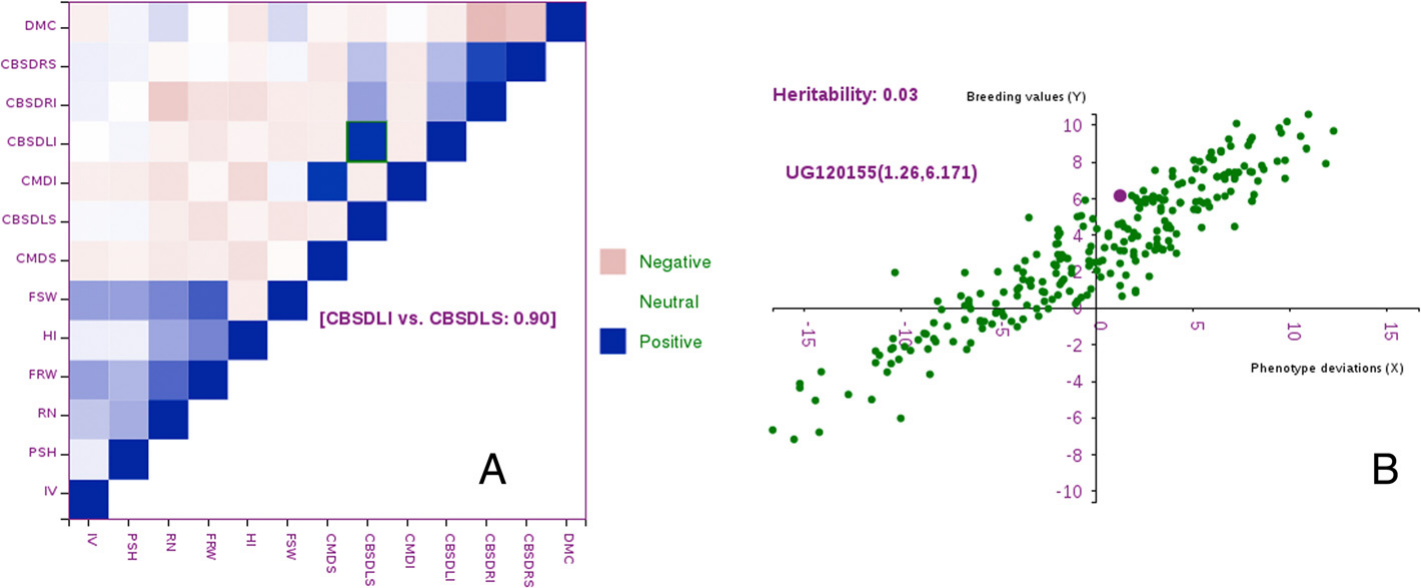
**Relationship plots.** Panel **A** shows an example phenotypic correlation among traits in a training population. Panel **B** shows the relationship between the GEBVs and phenotype values (as deviations from the mean) for a trait in a training population. Mousing over a data point in both plots shows the data for the corresponding coordinates.

In this scenario, breeders can search the database with the names of the traits of interest, one trait at a time, e.g. ‘dry matter content’, from the tool’s homepage (Additional file 1A). They will get a list of training populations and trials containing individuals with genotype data and that are phenotyped for the trait of their interest (Additional file 2). In principle, all individuals in a trial with phenotype and genotype data can be used to create the prediction model for the trait. Therefore, they can choose a trial or combination of trials, relevant to their target environment, and include all individuals in fitting the model. As a result, they will get the prediction model, its accuracy value, heritability of the trait, the GEBVs of all the individuals used in the model, additive genetic effects of each marker, and a list of relevant selection populations to which the model can be applied to predict their GEBVs for the trait. The GEBVs, visualized in a scatter plot, can be explored interactively by mousing over or zooming into the plot and downloaded in text format. This is demonstrated in Figure 1, which shows an example analysis output from a prediction model for the trait ‘dry matter content’ evaluated in a cassava training population called ‘NaCRRI Cassava Training Population’. Additional diagnostic outputs include descriptive statistics (not shown), scatter and frequency distribution plots of the phenotype data used in the model (Figure 2A,B) and scatter plot of the GEBVs against the phenotype values as deviations from the mean (Figure 3B).

#### Estimating GEBVs of selection candidates

*Suppose the breeders are further in their breeding programs and have selection candidates with genotype data only which are stored in the database. They want to estimate the breeding values of the selection candidates for the trait using a prediction model and advance superior performers to the next cycle of selection using the estimated breeding values.*

The first step is to choose a training population and generate a prediction model to use as described above in use case 1.1. Once on the prediction model web page, they will get a list of all relevant selection populations in the database composed of individuals associated with the training population. Only selection populations genotyped by markers matching the ones used to genotype the training population will be shown (Figure 1D). Additionally, if breeders have a custom list of selection candidates, they will programmatically also appear on the prediction model web page, when logged in to their user account (Figure 1E). To predict the breeding values of all the selection candidates for the trait, they click the population name or the ‘Predict’ link; a display of the trait name indicates the analysis result is ready, which can be viewed by following the link. The resulting GEBVs of the selection candidates are visualized in a scatter plot and the data can be viewed interactively by mousing over or zooming into the plot. The whole GEBVs dataset is also downloadable in text format.

### Use case 2: Trial approach

#### Creating a prediction model

*This approach is useful when breeders know their traits of interest were phenotyped in one or more trials of a training population. It can be used when they want to search for trials or training populations relevant to their target environment. This approach is also useful when breeders want to estimate GEBVs for multiple traits simultaneously.*

For this approach, breeders can browse and select a trial, a combination of trials, or existing training populations in the ‘Select a training population or create a new one using one or more trials’ section on the homepage of the tool (Additional file 1B). If they select multiple trials, individuals from all trials and with common traits phenotyped are combined. Next, a ‘training population’ webpage, with all traits phenotyped in the chosen trial or common traits in the case of combined trials will be shown (Additional file 3). From this webpage, they can select the trait(s) for which to fit prediction model(s). If they select a single trait, then they will obtain the same model output and workflow to predict GEBVs of selection candidates as shown in use case 1 (Figure 1).

**Figure 4:**
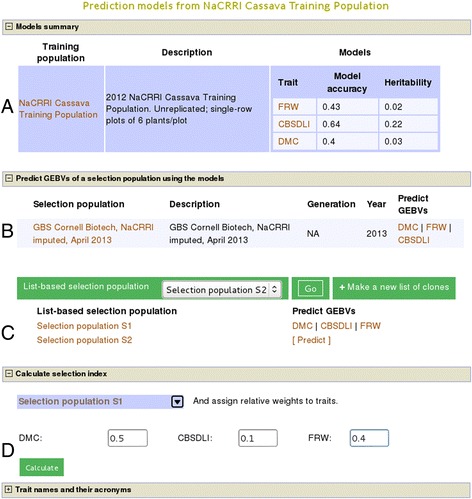
**Example of multiple prediction models output.** Panel **A** shows a list of models simultaneously fitted for multiple traits from a single training population (Additional file 2), with their correspoxnding accuracy and heritability of the traits. Detailed results of each model can be viewed by clicking the trait names (Figure 1). In panels **B** and **C** are lists of selection populations that the models can simultaneously be applied to estimate the GEBVs for the respective traits. Display of a trait name indicates the prediction for the trait is done. In panel **D**, the selection index calculator is shown for individuals, from training and selection populations, with GEBVs.

If breeders select multiple traits, e.g. traits ‘cassava brown streak disease leaf incidence’, ‘dry matter content’, and ‘fresh root weight’ from the ‘NaCRRI Cassava Training Population’ (Additional file 3), a prediction model for each trait will be created simultaneously. Each model’s summary and utility features are presented on a new web interface (Figure 4). On the webpage, a summary of each model including prediction accuracy, trait heritability and a link to the detail page of each model is displayed (Figure 4A). Following the links of each trait model, they will see in detail the respective model results and workflow as described in use case 1 (Figure 1).

Also on the multiple models page (Figure 4B,C), breeders will see selection populations on which the models can be applied to, section C is visible only when logged in. This enables them to simultaneously apply all the models on a selection population and estimate GEBVs of the selection candidates for each trait, which adds efficiency and flexibility to the GS process.

From the single trait model page, breeders can obtain the GEBVs of a trait and use it for tandem selection. However, they are most likely to select individuals based on their performance on multiple traits as revealed by a selection index. To facilitate this, when GEBVs are estimated for multiple traits, they can use a selection index calculator (Figure 4D) on the multiple models page. With this tool, they can assign relative weights to each trait, compute the selection index for each individual and download the result.

When planning to improve traits, it is important to know if there are correlations between the traits of importance. This can help in understanding how selection of one trait can influence the selection of another trait. Hence, phenotypic correlation coefficients (Figure 3A) are computed for traits evaluated in a trial and graphically presented. By mousing over coordinates in the correlation heatmap breeders can learn the strength and magnitude of the relationship between any pair of traits.

### Use case 3: Custom lists approach

*In the above two cases, when building a GS model a whole set of individuals from a trial or multiple trials is used. There are scenarios, however, when breeders may want to cherry-pick individuals evaluated in a trial or multiple trials and create a prediction model based on the custom list of select individuals. Alternatively, breeders may want to apply a prediction model and estimate GEBVs for a custom list of selection candidates.*

On Cassavabase, a ‘Lists’ tool (not shown) allows breeders to compose custom lists of individuals, which can be used for training and selection populations. The lists are stored in the users account and persist between log-ins. The individuals for a training population can be selected on an observation unit basis, e.g., their plot identifier, whereas for the selection candidates the genotype name can be used. To build a prediction model using a training population from a custom list as input, breeders can go to the ‘Select a list-based training population or create new’ section on the GS tool home page (Additional file 1C). Once they select a custom training population, a list of traits evaluated on the custom training set will be shown. They can then individually or simultaneously fit prediction model(s) for their selected trait(s). The rest of the workflow for this approach is as described in use case 2 for the trial approach.

Prediction of GEBVs for a custom list-based selection population is the same as for trial based selection populations as described above in use case 1 and 2. To estimate GEBVs of a custom list-based selection population, they can go to a relevant model(s) output page, where their custom selection population will be shown, and apply the model(s).

## Results and discussion

solGS makes GS data management, analysis, visualization and sharing user-friendly and efficient for breeders, as demonstrated using its implementation on the http://cassavabase.org/solgs. The application depends on a generic, flexible, data storage relational database schema that can make the tool relevant in any breeding program implementing the GS approach. Once relevant data is in the database, data analysis, visualization and sharing is a matter of point-and-click on an intuitively designed workflow.

The tool provides three approaches to choose a training population for fitting a prediction model. (1) With a trait in mind but little knowledge about what individuals were genotyped and phenotyped for the trait or in what trials they were phenotyped, breeders can search the database using the trait name and use the individuals in one or more relevant trials to build the prediction model for the trait. (2) Alternatively, they can browse the list of trials with phenotype and genotype data and select one or more trials relevant to their target environment or selection candidates. (3) Additionally, they can also compose and use a custom list of individuals, known to have phenotype and genotype data in the database. Approaches 2 and 3 are more efficient options in that breeders can (i) see all traits evaluated in the chosen trial(s) and study their correlation and thus decide efficiently on what traits to focus, (ii) build models for multiple traits at once, (iii) simultaneously apply multiple models on selection candidates to predict their GEBVs for the respective traits, and (iv) use the built-in selection index calculator.

Breeders at later stages of selection cycles predict GEBVs for their selection candidates by first deciding on what training population to use for the prediction model fitting as described above. Once the model(s) are generated, on the model page they will automatically see the relevant selection populations listed. A click on a selection population predicts and generates the GEBVs of the candidates using the model(s).

The graphical representation of data in the browser enables breeders to interactively explore the input and output of a model. The analysis result is also downloadable in text format.

Several statistical models are used for genomic prediction, including RR-BLUP, BayesA, BayesB [1], and Bayesian LASSO [20]. Lorenz et al. [21] have reviewed the differences among the models including their effects on prediction accuracy. Yi and Jannink [22] suggest a multivariate approach for genomic selection of multiple traits to improve prediction accuracy on low heritability traits genetically correlated to high heritability traits. Currently, the solGS tool implements a univariate RR-BLUP method, which is the most common method [9].

There are some public efforts to build bioinformatics infrastructure for GS. A United States Department of Agriculture (USDA) database stores only genotype data of dairy cattle from a single SNP array for use in genomic selection and animal tracking [23]. The Triticeae Coordinated Agricultural Project (T-CAP), USDA, is developing a web portal (http://triticeaetoolbox.org/) for accessing and analyzing GS data for barley and wheat generated by the project. An International Crops Research Institute for Semi-Arid Tropics (ICRISAT) project is also developing a desktop application called ISMU 2.0 for SNP and GS analysis with methods including RR-BLUP, Bayesian and Random Forest methods [24]. However, the application is for local use only and depends on the user’s computer file system for data storage. This limitation creates challenges in a long-term storage, community access, analysis and data sharing. Also often, project-centric web-portals that use custom-designed database schemas are difficult to adapt to new projects.

solGS relies on a generic, modular, flexible database schema for all GS data storage that can be employed for any organism. The schema is developed by a community of curators from several public databases and is already implemented by the Solanaceae Genomics Network [25], Cassavabase (http://cassavabase.org), Genome Database for Rosaceae (GDR; [26], Citrus Genome Database (www.citrusgenomedb.org), Cool Season Food Legume Genome Database [27], VectorBase [28] and KnowPulse (http://knowpulse2.usask.ca/portal/). Therefore, the solGS web application can be integrated easily into websites that use the ND database schema as backend for their data storage.

The application can serve as a medium for community data and knowledge exchange, similar to the functioning of the SGN community annotation [29] and QTL analysis and linking to genomes tools [30]. Depending on data access privileges, solGS can facilitate web access and exchange of data on breeding material among a community of researchers. Sharing GS output can be done conveniently through exchanging model output page links or data downloads.

In the near future, we plan to integrate more features into the application to enhance the decision-making efficiency and capability of GS breeders. We will calculate superior progeny values of individuals based on expected mean values of progenies, expand the univariate RR-BLUP modeling into multivariate analysis, and run genetic correlation analysis and principal component analysis of individuals based on their genotypes. Depending on the availability of R packages, we will add more modeling options such as the Bayesian methods and supervised classification algorithms. We will add algorithms to preprocess phenotype data from experimental designs newly added to the database. We will write a comprehensive user manual and tutorials. To speed up the prediction process, we will parallelize analyses.

## Conclusions

solGS is a web-based tool for genomic selection. It has an intuitive workflow for choosing a training population on which to fit a prediction model and estimating GEBVs of selection candidates. Model input and output is visualized graphically and can be interactively explored or downloaded in text format. Its dependence on the generic, flexible, Chado ND database schema, for its data storage system, makes the tool adaptable to wide range of GS breeding programs.

## Availability and requirements

- **Project name:** solGS, Genomic selection tool.
- **Project home page:**http://github.com/solgenomics; http://cassavabase.org/solgs.
- **Operating system(s):** Platform independent.
- **Programming language:** R, Perl, Mason, JavaScript, D3
- **Other requirements:** Internet connection, a browser.
- **Any restrictions to use by non-academics:** None.

## Additional files

Additional file 1:
**solGS homepage: web-interfaces for choosing a training population to create a genomic prediction model.** From the tool’s homepage breeders decide what individuals to use in their models in three ways. The first method, shown in panel A, uses a trait name to search the database for individuals phenotyped for that trait and select the individuals from any number of trials (Additional file 2). A second way (panel B) is to search for a training population or trials of interest and use the set of individuals evaluated in a trial or combination of trials. A third way (panel C) is to make a custom list of individuals using Cassavabase’s ‘List’ feature. Additional file 2:
**Example of a list of trials in all of which a given trait was phenotyped.** When breeders search using a trait name for phenotyped individuals to create a training population and use in a prediction model, they get a list of relevant training populations or trials. All Individuals from a trial or combination of trials can be used. Additional file 3:
**Example of training population detail page.** From a training population’s page, breeders can select any number of traits and simultaneously fit models for them. They can also study the phenotypic correlation among the traits (Figure 3A).
