# Supplementary figures and images for: solGS: a web-based tool for genomic selection
Source: BMC Bioinformatics. 2014 Dec 14;15(1):398. doi: 10.1186/s12859-014-0398-7 (PMC4269960; doi:10.1186/s12859-014-0398-7)

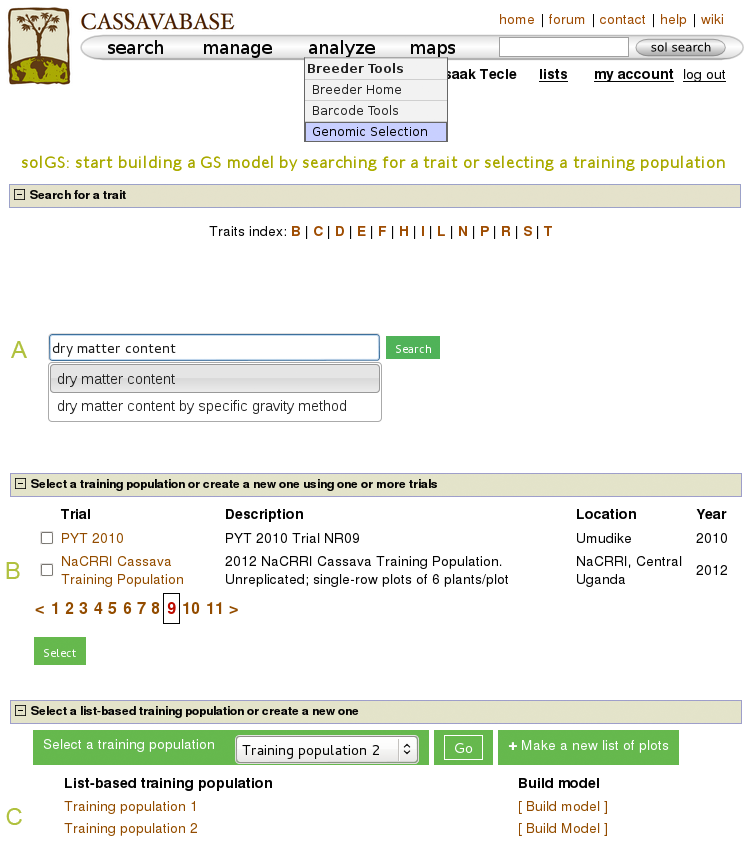

Supplement: Additional file 1: — solGS homepage: web-interfaces for choosing a training population to create a genomic prediction model. From the tool’s homepage breeders decide what individuals to use in their models in three ways. The first method, shown in panel A, uses a trait name to search the database for individuals phenotyped for that trait and select the individuals from any number of trials (Additional file 2). A second way (panel B) is to search for a training population or trials of interest and use the set of individuals evaluated in a trial or combination of trials. A third way (panel C) is to make a custom list of individuals using Cassavabase’s ‘List’ feature. [file 12859_2014_398_MOESM1_ESM.png]

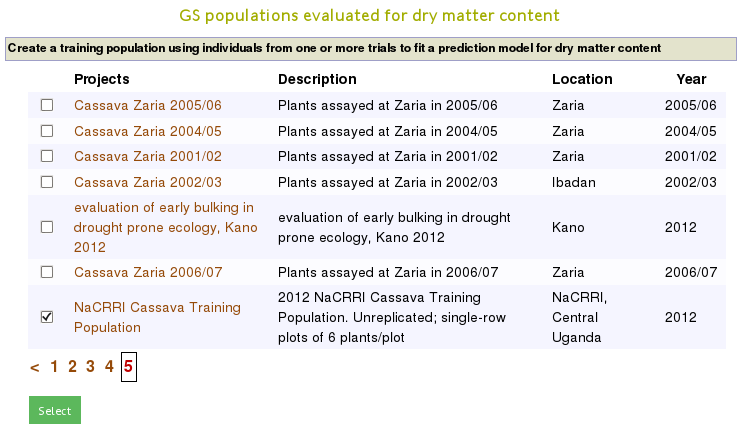

Supplement: Additional file 2: — Example of a list of trials in all of which a given trait was phenotyped. When breeders search using a trait name for phenotyped individuals to create a training population and use in a prediction model, they get a list of relevant training populations or trials. All Individuals from a trial or combination of trials can be used. [file 12859_2014_398_MOESM2_ESM.png]

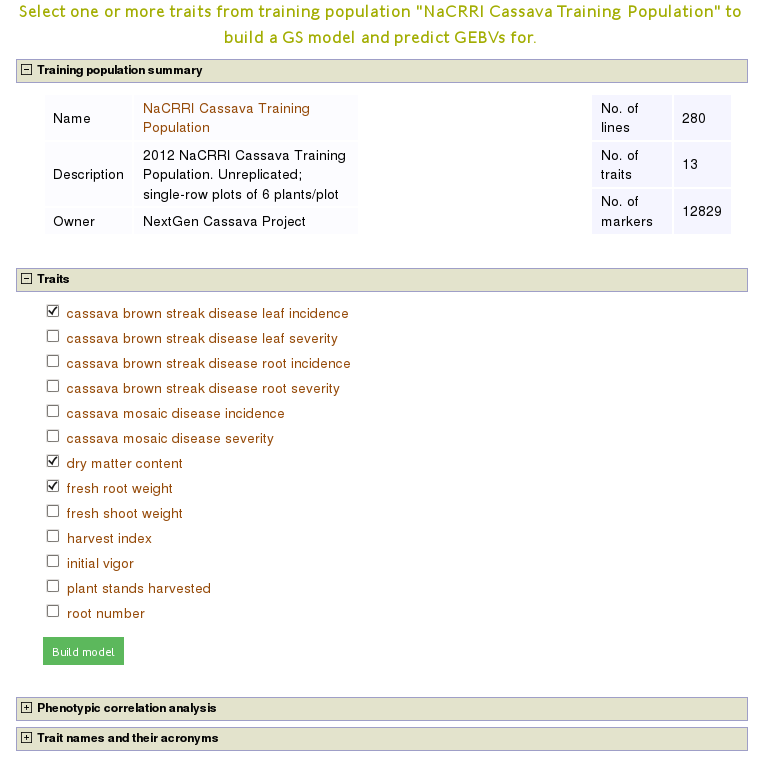

Supplement: Additional file 3: — Example of training population detail page. From a training population’s page, breeders can select any number of traits and simultaneously fit models for them. They can also study the phenotypic correlation among the traits (Figure 3A). [file 12859_2014_398_MOESM3_ESM.png]
